# The Effect of Light Sedation with Midazolam on Functional Connectivity of the Dorsal Attention Network

**DOI:** 10.3390/brainsci11081107

**Published:** 2021-08-22

**Authors:** Junkai Wang, Yachao Xu, Gopikrishna Deshpande, Kuncheng Li, Pei Sun, Peipeng Liang

**Affiliations:** 1 Department of Psychology, Tsinghua University, Haidian District, Beijing 100084, China; plzgg@mail.tsinghua.edu.cn; 2 Department of Anesthesiology, Xuanwu Hospital, Capital Medical University, Beijing 100053, China; xuyachao@xwhosp.org; 3 School of Psychology, Capital Normal University, Haidian District, Beijing 100048, China; gopi@auburn.edu; 4 Beijing Key Laboratory of Learning and Cognition, Capital Normal University, Beijing 100048, China; 5 AU MRI Research Center, Department of Electrical and Computer Engineering, Auburn University, Auburn, AL 36849, USA; 6 Department of Psychological Sciences, Auburn University, Auburn, AL 36849, USA; 7 Alabama Advanced Imaging Consortium, Birmingham, AL 35233, USA; 8 Center for Neuroscience, Auburn University, Auburn, AL 36849, USA; 9 Department of Psychiatry, National Institute of Mental Health and Neurosciences, Bangalore 560030, India; 10 Center for Brain Research, Indian Institute of Science, Bangalore 560012, India; 11 Department of Radiology, Xuanwu Hospital, Capital Medical University, Beijing 100053, China; likuncheng@xwhosp.org; 12 Beijing Key Laboratory of Magnetic Resonance Imaging and Brain Informatics, Capital Medical University, Beijing 100053, China

**Keywords:** resting-state fMRI, between-network anticorrelation, dorsal attention network, midazolam, sedation

## Abstract

Altered connectivity within and between the resting-state networks (RSNs) brought about by anesthetics that induce altered consciousness remains incompletely understood. It is known that the dorsal attention network (DAN) and its anticorrelations with other RSNs have been implicated in consciousness. However, the role of DAN-related functional patterns in drug-induced sedative effects is less clear. In the current study, we investigated altered functional connectivity of the DAN during midazolam-induced light sedation. In a placebo-controlled and within-subjects experimental study, fourteen healthy volunteers received midazolam or saline with a 1-week interval. Resting-state fMRI data were acquired before and after intravenous drug administration. A multiple region of interest-driven analysis was employed to investigate connectivity within and between RSNs. It was found that functional connectivity was significantly decreased by midazolam injection in two regions located in the left inferior parietal lobule and the left middle temporal area within the DAN as compared with the saline condition. We also identified three clusters in anticorrelation between the DAN and other RSNs for the interaction effect, which included the left medial prefrontal cortex, the right superior temporal gyrus, and the right superior frontal gyrus. Connectivity between all regions and DAN was significantly decreased by midazolam injection. The sensorimotor network was minimally affected. Midazolam decreased functional connectivity of the dorsal attention network. These findings advance the understanding of the neural mechanism of sedation, and such functional patterns might have clinical implications in other medical conditions related to patients with cognitive impairment.

## 1. Introduction

Conscious sedation represents an initial stage of anesthesia accompanying alteration of consciousness [1]. Despite being widely used for nearly two centuries in clinical practice, the neural mechanism by which anesthetics induce altered consciousness is still incompletely understood [2]. Resting-state functional magnetic resonance imaging (rs-fMRI) is a powerful and fundamental tool for the noninvasive mapping of spontaneous neuronal activity and functional interactions between several large-scale networks in the brain [3,4,5]. Numerous neuroimaging studies have indicated that low frequency fluctuations of blood oxygenation level-dependent (BOLD) fMRI signal during the resting-state is related to spontaneous brain activity [6,7]. Accumulating evidence has indicated that activity and connectivity in lower-level networks, including the sensorimotor network (SMN), auditory network (AN), and visual network (VN) appear to persist at reduced levels of consciousness: Brain activation declines first in higher-order brain networks, including the salience network, default mode network (DMN), and executive control network (ECN) before responses in primary sensory cortices are attenuated [8,9,10,11].

There is now indisputable evidence that anesthetics modify higher-order resting-state networks (RSN) [8,12,13]. However, anesthesia alters brain activity and regional cerebral metabolism in a dose-dependent manner, and different anesthetic agents may produce a variety of functional connectivity patterns [8,14]. Functional connectivity changes during anesthetic-induced light sedation are still unclear, and RSNs that may be sensitive to alterations in states of consciousness still require further investigation. According to previous literature, consciousness is typically classified into two aspects: level and content [15,16,17]. The level of consciousness is usually referred to as the degree of wakefulness or arousal, while the content of consciousness refers to awareness [15,16]. During anesthetic-induced alteration of consciousness, anesthesia may produce different consciousness states, including awareness of the environment, disconnection from the environment and only awareness of self, and unconsciousness, depending on the anesthetic agent and dose [18,19]. Thus, participants may experience a state from normal conscious wakefulness to a state of decreased awareness of the environment during anesthetic-induced light sedation [15,19]. Awareness of the environment, which is also defined as external awareness, requires the sensory modalities for environmental perception, whereas awareness of self, which is also defined as internal awareness, is a mental process that focuses on one’s internal state and does not require the sensory input [20]. Considering neuroimaging evidence, awareness may depend on two main RSNs: a dorsal attention network (DAN) routinely mediating external processing and attention-demanding cognitive function and a DMN, which is engaged during self-related processes [21,22]. Although numerous studies have repeatedly shown the effect of anesthetic agents on the functional connectivity of the DMN, the results are still inconsistent. Under anesthesia, both decreased connectivity and increased connectivity within DMN have been reported [9,23,24,25]. In our previous study, which was restricted to the functional connectivity within different resting-state networks, we also did not identify significant connectivity changes within the DMN during midazolam-induced light sedation [26]. In addition, changes in functional connectivity within the DAN are still poorly investigated, hampering our understanding of the neural basis of the effects of midazolam-induced light sedation on external awareness.

It is well established that the anticorrelation between the DAN and the DMN (i.e., negative DAN connectivity with DMN) is important for normal cognitive abilities, suggesting that an increase in anticorrelation indicates an increase in capacity to switch between internal and external modes of attention and flexibly allocate attentional resources [27]. Thus far, decreased DAN–DMN anticorrelation has been reported during propofol-induced loss of consciousness and ketamine-induced alterations of consciousness [9,13]. Moreover, the DAN–DMN anticorrelation has been reported to be a more reliable marker for tracking alterations in a one’s state of consciousness than connectivity within the DMN in patients with disorders of consciousness [28]. However, it is yet unclear how this anticorrelation changes during midazolam-induced light sedation (i.e., whether the anticorrelation effects on external awareness observed with other anesthetics are generalizable to midazolam as well as the effects of light sedation, as opposed to deep sedation).

In the current study, we aimed to examine connectivity within the DAN and DAN–DMN anticorrelation changes during midazolam-induced light sedation. A placebo-controlled, cross-over, within-subjects experimental design was adopted in this study, with which the sedation effect of interest could be separated from the sham effects induced by the injection measurements (pre-injection measurements versus post-injection measurements). Based on aforementioned findings, it was hypothesized that diminished functional connectivity of the DAN might be observed during midazolam-induced light sedation.

## 2. Materials and Methods

### 2.1. Participants

Fourteen healthy right-handed volunteers (8 males and 6 females) participated in the study (aged 20 to 30 years; mean age ± SD, 24 ± 3.2 year). Exclusion criteria for all participants included history of head trauma or any surgery or procedure that involved anesthesia, drug addiction, history of medical, neurological or psychiatric disease, prescription use of benzodiazepines or benzodiazepine-like hypnotics, or any of the contraindications for an MRI scan, such as vascular clips or metallic implants. Moreover, all volunteers were required to refrain from alcohol and caffeine for at least 12 h prior to being scanned. The experimental procedure was approved by the Institutional Review Boards (IRBs) of Xuanwu Hospital and Capital Medical University. Written informed consent was obtained from all volunteers after the study had been fully explained.

**Figure 1:**
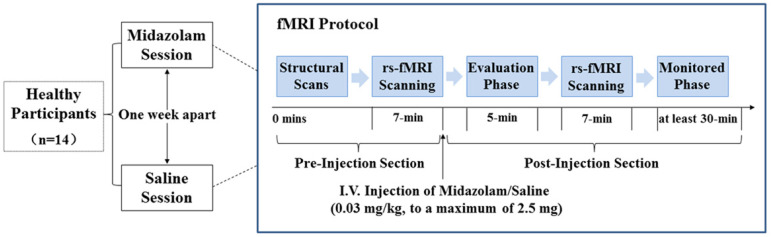
Study procedures of the study. Abbreviations: rs-fMRI, resting-state functional magnetic resonance imaging; I.V., intravenous injection.

The data set included in this analysis was previously published by Liang et al. [26]. The details on the experimental design are as follows. The study was a double-blind, cross-over, within-subjects design. As illustrated in Figure 1, volunteers had randomly received either midazolam (MZ) or saline (SA) in two sessions with a 1-week interval. Each session consisted of a pre-injection and a post-injection imaging section.

Prior to entering the scanner, saline was infused at 100 mL/h to maintain infusion through a 22-gauge intravenous cannula. For the pre-injection section, the structural MRI data were first acquired, which was then followed by a 7 min rs-fMRI scanning. After that, participants were given an intravenous injection of either midazolam (0.03 mg/kg of the participant’s body mass, to a maximum of 2.5 mg) diluted to a total volume of 10 mL or a matching volume of saline through the intravenous cannula within a 2 min period [26,29]. The sedation level of volunteers was estimated using a six-point modified Observer Assessment of Alertness and Sedation scale (OAA/S, [30]) ranged from 0 to 5. In this study, all volunteers got an OAA/S of 4 in the midazolam session (i.e., volunteer makes slow response to name spoken in normal tone/sleeping when undisturbed). After drug injection, there was a time interval of about 5 min before scanning, allowing for evaluation of the sedation level, verification of volunteers’ safety, and midazolam concentration reaching a steady state. Then, a 7 min rs-fMRI scanning was performed in the post-injection section. During the whole experimental session, vital signs were continuously monitored using an MR-compatible monitor. After scanning, volunteers were monitored for at least 30 min until they had completely recovered. Then, the intravenous cannula was removed, and the monitor was disconnected.

### 2.2. Image Preprocessing

Preprocessing and connectivity analyses were conducted using a statistical parametric mapping software package (SPM 8, http://www.fil.ion.ucl.ac.uk/spm/software/spm8, accessed on 25 April 2019) and the CONN-fMRI functional connectivity toolbox v18b (http://www.nitrc.org/projects/conn, accessed on 17 May 2019; [31]). The first 10 volumes of the functional images were discarded to allow for signal stabilization and the participants’ adaptation to the noisy environment. The remaining 200 volumes were slice-time corrected (to the middle slice) and then realigned to the first volume to correct for inter-scan head motions. No participant had head motion of more than 2 mm in translation or 2° in rotation. We also calculated motion parameters (estimated by a realignment algorithm) for each group, and the difference between groups was statistically analyzed. The motion-corrected functional volumes were co-registered to the T1 structural image and spatially normalized to the Montreal Neurological Institute (MNI) space (re-sampled to 3 mm isotropic voxels). Subsequently, the functional images were spatially smoothed by a Gaussian filter with a full-width half-maximum (FWHM) of 6 mm.

Subsequent steps were performed using the CONN toolbox. As functional connectivity is influenced by head motion, we used the artifact detection tool (ART) as implemented in the CONN functional connectivity toolbox to detect the motion outliers (artifacts). Specifically, an image was defined as an outlier image if the head motion was more than 2 mm in translation or 0.02 radians in rotation from the previous frame. Outliers were subsequently included as nuisance regressors (i.e., one regressor per outlier within the first-level general linear model). Therefore, the temporal structure of the data was not disrupted. Then, spurious sources of noise were estimated and regressed out using the anatomical component base noise reduction strategy (aCompCor; [32]). Global signal regression was not used because it can artificially introduce anticorrelations into the BOLD signal [33]. The motion parameters and their first-order temporal derivatives were also regressed out. Finally, the time series were filtered with the recommended bandpass filter [34], followed by removing the linear trend.

### 2.3. Functional Connectivity Analysis

A multiple region of interest (ROI)–driven analysis was employed to investigate within and between RSNs’ connectivity. This ROI-driven analysis was described in previous studies and considered to have the advantage of characterizing brain networks in volunteers [13,35]. Based on previous evidence, there is a common consensus that connectivity in lower-level networks such as the SMN is preserved at reduced levels of consciousness [8,9,26]. Therefore, the functional connectivity of the SMN was employed as a reference to examine the repeatability and reliability of the results in this study. Peak coordinates corresponding to multiple seed regions within the DAN and the SMN were selected from the literature [36,37]. ROIs for the DAN included regions centered in the intraparietal sulcus (IPS; MNI coordinates: –34, –38, 44), the frontal eye field (FEF; MNI coordinates: –22, –8, 54), the ventral frontal region precentral ventral (PrCv; MNI coordinates: –49, 3, 34), the middle temporal motion complex (MT; MNI coordinates: –51, –64, –2), the anterior superior parietal lobule (SPL7a; MNI coordinates: –18, –69, 51), and the posterior superior parietal lobule (SPL7p; MNI coordinates: –8, –63, 57). For the SMN, ROIs were located in the left and right primary motor cortices (LPrMC/RPrMC; MNI coordinates: −39, −26, 51/38, −26, 48) and the supplementary motor areas (SMA; MNI coordinates: 0, −21, 48). For each network, time series from the voxels in each seed region (defined as 6 mm radius spheres around peak coordinates) were extracted and then averaged together. Whole-brain positive and negative correlation maps were estimated on the basis of fMRI time series, and Pearson’s correlation analysis was performed between averaged time series of the seed ROIs ascribed above and all other voxels in the brain. Then, correlation maps were converted to *z*-value maps using Fisher’s *r*-to-*z* transformation to improve the normality of the correlation coefficients and allow for group-level comparisons.

### 2.4. Group-Level Connectivity Analysis

To explore the sedation effect of interest on functional connectivity of the SMN and DAN, the spatial maps of both networks were determined first. One-sample *t*-tests were performed on all volunteers’ individual *z*-value maps for pre-injection phase (pre-midazolam or pre_MZ and pre-saline or pre_SA, respectively). Binary maps were generated to create a reference to ensure proper network characterization with a cluster-level FWE correction of *p* < 0.05 and served as a mask for subsequent analyses. Then, statistical analysis was conducted for group differences using 2 × 2 two-way repeated measures analysis of variance (ANOVA) on all volunteers’ individual correlation maps for pre_MZ (pre-midazolam administration), pre_SA (pre-saline administration), post_MZ (post-midazolam administration), and post_SA (post-saline administration). Two main effects, drug and injection measurements (pre-injection measurements versus post-injection measurements), as well as an interaction (drug × injection measurements) effect were obtained by using clusters with a minimum size of 10 voxels and an individual voxel height threshold of *p* < 0.001, masked with spatial maps of the DAN or SMN. Regions from the ANOVA that survived the threshold subsequently served as ROIs for revealing the effect of the interaction (drug-by-injection measurements). Specifically, the pre_MZ and post_MZ differences and the pre_SA and post_SA differences were reported to observe which group drove the effect. We were only interested in the interaction effect, which represents the true drug effect. The mean functional connectivity scores (*z*-values) were extracted from clusters with a significant interaction effect among volunteers under each condition. The *z*-values within the clusters for pre_MZ, pre_SA, post_MZ, and post_SA were plotted using GraphPad Prism 6 (GraphPad Software, Inc., (San Diego, CA, USA) www.graph-pad.com, accessed on 26 August 2019) for visualization.

## 3. Results

### 3.1. Head-Motion Estimation

The head motion index was used to assess the possible differences between pre-injection and post-injection runs (midazolam or saline). The average framewise displacement (FD) was calculated for each individual under different conditions [38]. Comparing the average FD values across different conditions using two-way repeated measures ANOVA, no differences in motion displacement were found among the four conditions (*p* > 0.05).

### 3.2. Functional Connectivity Analysis

#### 3.2.1. SMN Connectivity Results

**Figure 2:**
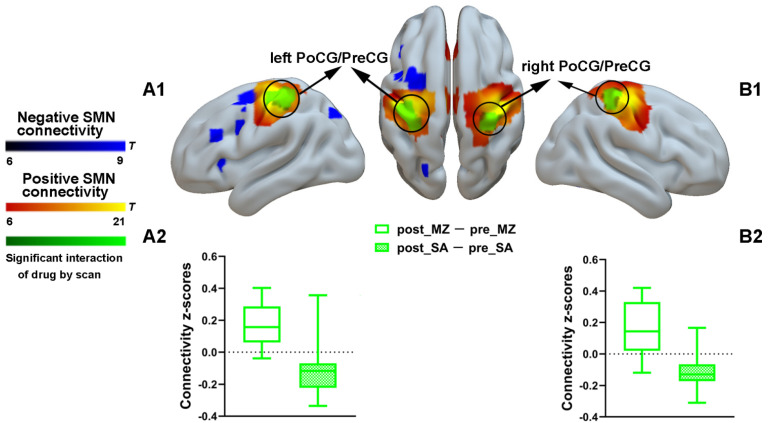
Spatial differences in functional connectivity of the SMN by midazolam injection. Two-way repeated-measures ANOVA revealed significantly increased positive functional connectivity (in green) by midazolam injection between regions predefined as pertaining to SMN and the left postcentral gyrus and precentral gyrus (**A1**) and right postcentral gyrus and precentral gyrus (**B1**). Significant results are overlaid on the spatial maps (cluster-level FWE correction) of positive SMN connectivity (shown in warm yellows) and negative SMN connectivity (shown in blue). (**A2**) Functional connectivity of the post_MZ-pre_MZ and the post_SA-pre_SA within the left postcentral gyrus and precentral gyrus cluster of the SMN that showed significant interaction between drug and injection measurements among volunteers under each condition. (**B2**) Functional connectivity of the post_MZ-pre_MZ and the post_SA-pre_SA within the right postcentral gyrus and precentral gyrus cluster of the SMN that showed significant interaction between drug and injection measurements among volunteers under each condition. Boxplot whiskers indicate min and max. Box is defined by 25th percentile, median, and 75th percentile. Bars represent average functional connectivity strength (*z*-values), and error bars indicate standard error (SE) derived from the ANOVA test on the cluster that showed significant interaction between drug and injection measurements. Abbreviations: PoCG, postcentral gyrus; PreCG, precentral gyrus; SMN, sensorimotor network; MZ, midazolam; SA, saline.

To assure repeatability and reliability of the results in the current study, a spatial map of the SMN was obtained to characterize the positive and negative SMN connectivity (Supplementary Figure S1). Only positive SMN connectivity was significantly increased by midazolam injection, solid evidence of reduced levels of consciousness, as has been shown in previous studies [23,26]. Specifically, during midazolam injection, relative to the saline treatment baseline, functional connectivity was significantly enhanced between regions predefined as pertaining to SMN located in the bilateral postcentral gyrus and precentral gyrus (Figure 2 and Supplementary Table S1).

#### 3.2.2. The Spatial Distribution of the DAN and DMN

**Figure 3:**
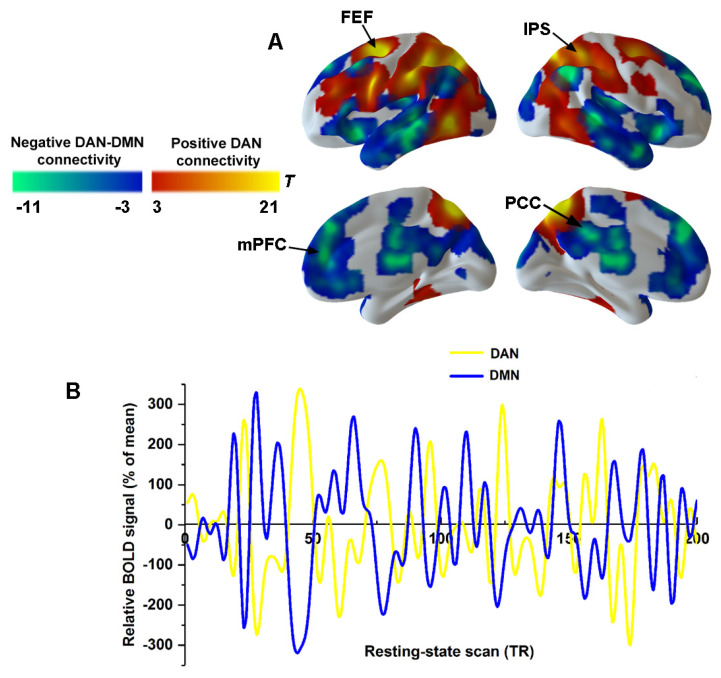
Anticorrelation between DAN and DMN. (**A**) Seed-based functional connectivity for pre-injection section was used to derive spatial masks for DAN (warm color) and DMN (cold color). (**B**) Single volunteer time series are illustrated for DAN connectivity (yellow) and DMN connectivity (blue). Abbreviations: PCC, posterior cingulate cortex; mPFC, medial prefrontal cortex; IPS, intraparietal sulcus; FEF, frontal eye fields; DAN, dorsal attention network; DMN, default mode network.

To characterize the connectivity within the DAN and DAN–DMN anticorrelation, the spatial distribution of the DAN and DMN was obtained by using correlational maps for pre-injection sections (Figure 3A). The IPS and FEF showed the most robust positive connectivity within the DAN (Figure 3A). The posterior cingulate cortex (PCC) and the medial prefrontal cortex (mPFC) revealed strong negative connectivity with the DAN, which actually represented the DMN (Figure 3A). Figure 3B shows the time courses of a single volunteer for the DAN (yellow) and DMN (in blue), which replicated a classical anticorrelation pattern between the DAN and DMN.

#### 3.2.3. DAN Connectivity Results

**Figure 4:**
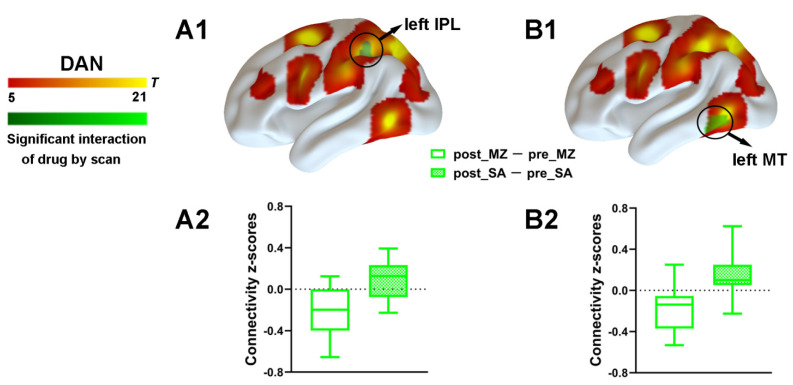
Spatial differences in functional connectivity within the DAN by midazolam injection. Two-way repeated measures ANOVA revealed significantly decreased positive functional connectivity (in green) by midazolam injection between regions predefined as pertaining to DAN and the left IPL (**A1**) and left MT (**B1**). Significant results are overlaid on the spatial maps (shown in warm yellows) of the DAN (cluster-level FWE correction). (**A2**) Functional connectivity of the post_MZ-pre_MZ and the post_SA-pre_SA within left IPL of the DAN that showed significant interaction between drug and injection measurements among volunteers under each condition. (**B2**) Functional connectivity of the post_MZ-pre_MZ and the post_SA-pre_SA within left MT of the DAN that showed significant interaction between drug and injection measurements among volunteers under each condition. Boxplot whiskers indicate min and max. Box is defined by 25th percentile, median, and 75th percentile. Bars represent average functional connectivity strength (*z*-values), and error bars indicate standard error (SE) derived from the ANOVA test on the cluster that showed significant interaction between drug and injection measurements. Abbreviations: IPL, inferior parietal lobule; MT, middle temporal area; DAN, dorsal attention network; MZ, midazolam; SA, saline.

**Figure 5:**
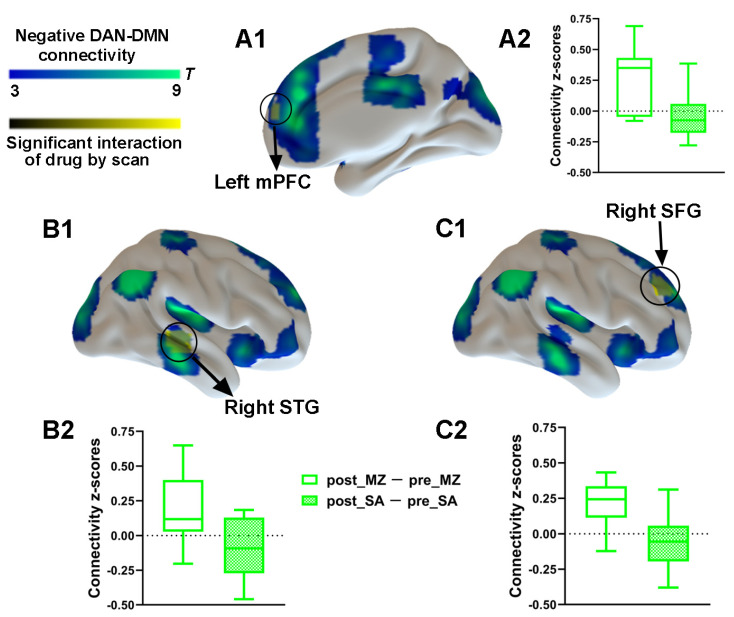
Spatial differences in functional connectivity between-network anticorrelations by midazolam injection. Two-way repeated-measures ANOVA revealed significantly decreased DAN–DMN anticorrelation (in yellow) by midazolam injection between regions predefined as pertaining to DAN and the left mPFC (**A1**) and right STG (**B1**) and right SFG (**C1**). Significant results are overlaid on the spatial maps (shown in cold blues) of the DMN (cluster-level FWE correction). (**A2**) Functional connectivity of the post_MZ-pre_MZ and the post_SA-pre_SA between left mPFC and DAN that showed significant interaction between drug and injection measurements among volunteers under each condition. (**B2**) Functional connectivity of the post_MZ-pre_MZ and the post_SA-pre_SA between right STG and DAN that showed significant interaction between drug and injection measurements among volunteers under each condition. (**C2**) Functional connectivity of the post_MZ-pre_MZ and the post_SA-pre_SA between right SFG and DAN that showed significant interaction between drug and injection measurements among volunteers under each condition. Boxplot whiskers indicate min and max. Box is defined by 25th percentile, median, and 75th percentile. Bars represent average functional connectivity strength (*z*-values), and error bars indicate standard (SE) derived from the ANOVA test on the cluster that showed significant interaction between drug and injection measurements. Abbreviations: mPFC, medial prefrontal cortex; STG, superior temporal gyrus; SFG, superior frontal gyrus; DAN, dorsal attention network; DMN, default mode network; MZ, midazolam; SA, saline.

To explore the effect of drug and injection measurements on functional connectivity, the main effects and interaction effect associated with the DAN was further examined by using the aforementioned spatial maps in a two-way ANOVA. A significant main effect of drug for the DAN connectivity was identified (Supplementary Figure S2). Most important, a significant interaction between drug and injection measurements was found. As compared with the saline condition, functional connectivity within the DAN was significantly decreased by midazolam injection located in the left IPL and the left MT (Figure 4 and Supplementary Table S1). Contrasting midazolam injection and the saline condition, three clusters in DAN–DMN anticorrelation for the interaction effect which included the left mPFC, the right STG, and the right SFG were also identified (Figure 5 and Supplementary Table S1). Connectivity between all regions and DAN was significantly decreased by midazolam injection.

**Figure 6:**
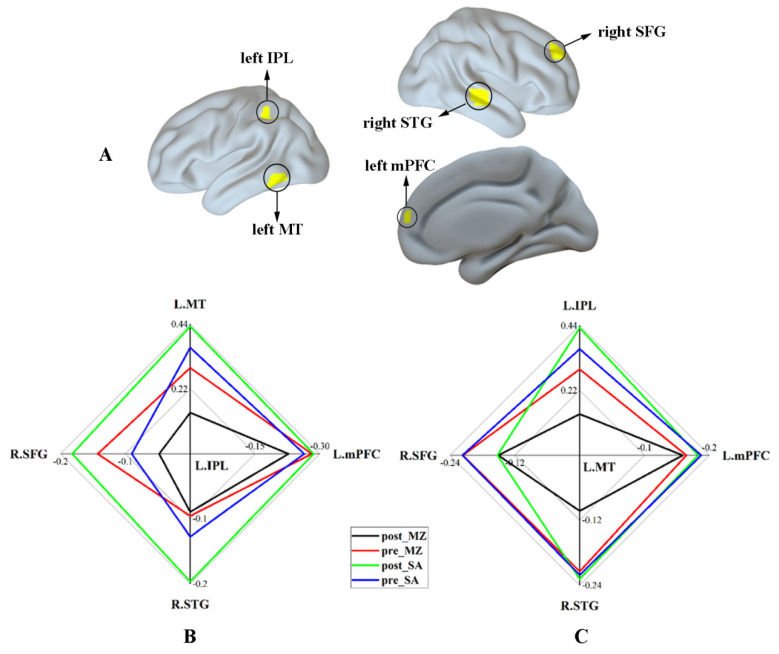
The relative strength of functional connectivity for the regions with a significant interaction effect. (**A**) The regions that revealed a significant interaction effect within DAN and DMN. (**B**) The functional connectivity strength between the left IPL and other regions with a significant interaction effect under different conditions. (**C**) The functional connectivity strength between the left MT and other regions with a significant interaction effect under different conditions. Abbreviations: IPL, inferior parietal lobule; MT, middle temporal area; mPFC, medial prefrontal cortex; STG, superior temporal gyrus; SFG, superior frontal gyrus; MZ, midazolam; SA, saline.

Finally, the significant interaction effect for functional connectivity associated with DAN was summarized. Figure 6A shows the regions that revealed a significant interaction effect within the DAN and DMN. Figure 6B,C shows the functional connectivity strength between regions within the DAN (including the left IPL and left MT) and the regions anticorrelated with the DAN (DMN; including the left mPFC, the right STG, and the right SFG) under different conditions, respectively. These results indicate reduced functional connectivity of the DAN by midazolam injection, as contrasted with the saline injection. Additionally, the different modulation effects of midazolam on the brain caused by sex hormone levels were not found in this study (Supplementary Figure S3).

## 4. Discussion

In the current study, first, we replicated the result that reactivity in lower-level networks (e.g., SMN) was intact or even enhanced at reduced levels of consciousness. Then, we detected reduced connectivity within the DAN and anticorrelation between the DAN and subregions of DMN during midazolam-induced light sedation, a state characterized by a reduced level of consciousness. These findings deepen our understanding of the neural mechanism of light sedation, specifically, DAN-mediated external awareness in it.

Consistent with prior studies, our results show increased within-network connectivity for the lower-level networks (e.g., SMN) during midazolam-induced light sedation [23,26,39]. It is still unclear why the lower-level networks increased their activity during sedation. A possible explanation is that enhanced functional connectivity within lower-level functional network may be related to a kind of compensation mechanism [26]. This was further supported by the analysis of amplitude of the low-frequency fluctuation (ALFF) [40], which showed that mean ALFF in the right supplementary motor area and the bilateral postcentral gyrus and precentral gyrus within the SMN was significantly increased by midazolam injection (Supplemental Figure S4 and Table S2). Neural systems autonomously compensate for the drug effect as a direct reaction to the drug injection, which helps to maintain information processing abilities [26]. However, the overcompensation elevates the baseline of within-network functional connectivity and may leave little reserve of capacity available if faced with additional cognitive demands. Thus, the higher basal threshold inhibits the brain response to the complex information processing by increasing the within-network connectivity.

As expected in our hypothesis, we found that functional connectivity within DAN was significantly decreased during midazolam-induced light sedation. Optimal attention performance in humans depends on the attention network. The DAN includes the dorsal fronto-parietal areas, such as the inferior parietal lobule and the middle temporal area, contributing to mediation of goal-directed processes and to orientation to external stimuli with rapid strategic control over attention [22]. Specifically, left IPL is a critical substrate for conceptual knowledge which requires the integration of information from multiple modalities including high-level visual and attentional processes for visual attention-demanding actions [41,42]. The left MT links to processing of visual motion information that is used to construct the motor commands and is related to the velocity of visual stimuli, which is critical to responding to visual cues [43]. Together with left IPL, these areas contribute to the normal visual attention and attention-demanding cognitive function. As mentioned before, awareness is an essential aspect of consciousness, which can be further divided into awareness of the environment (the extrinsic system) and awareness of self (the intrinsic system) [20]. The extrinsic system encompasses lateral fronto-parietal areas and is activated during goal-directed behavior [20]. Thus, the DAN should be involved in the extrinsic system and response to environmental cues through the sensory modalities.

The current results of reduced functional connectivity within higher-order brain networks have also been found in healthy individuals with alterations of consciousness induced by other hypnotic anesthetic agents, including propofol, sevoflurane, and ketamine [9,12,13], as well as in patients with disorders of consciousness [44]. These findings consistently indicate that diminished functional connectivity within higher-order networks may be responsible for reduced cognitive abilities observed under sedation/anesthesia, which represents a common feature of anesthesia-induced alteration of consciousness [9,12,13]. Doses of midazolam producing conscious sedation are associated with diminished external awareness, corresponding to slowing down of orienting to external stimuli. This explains volunteers’ longer response time.

It should be further mentioned that there is no consensus regarding the impact of sedation and anesthesia on functional connectivity within DMN. The different levels of anesthesia/sedation and different anesthetic agents may partially contribute to differences in the observed results. Moreover, accumulating evidence has proved that anesthetic agents affect different brain circuits in a heterogeneous manner [25,45]. Like locally organized lower-level perceptual networks, the brain regions within the DMN display strong structural connections that make it more persistent under anesthesia and seem to be minimally altered during light sedation [46]. This result is in line with previous studies showing that DMN connectivity is preserved in anesthetized monkeys and in people with unresponsive wakefulness syndrome [47,48]. Taken together, the aforementioned findings suggest that, relative to the DMN connectivity, functional connectivity associated with DAN might be more sensitive to alteration in the state of awareness of the environment. Moreover, the dorsolateral prefrontal cortex (a central hub of the DAN) showed the greatest decrease in activation during anesthetic-induced unconsciousness [12]. This observation also supports our assumption of a prominent role of the DAN in sedation through attentional mechanisms.

Consistent with our hypothesis, we also found that anticorrelation between the DAN and subregions of DMN was significantly decreased during midazolam-induced light sedation. The MPFC is a core hub of the DMN and plays a crucial role in attention, inhibitory control, working memory, spatial memory, and long-term memory [49]. Attending the attentional processing, the MPFC might be prominently involved in accurately responding to visual cues and inhibition of inappropriate responses [50]. Decreased anticorrelation between the DAN and the left MPFC might represent reduced efficiency of processing external visual cues and responding inappropriately to one’s surrounding environment. Additionally, growing evidence supports the idea that increased anticorrelation between the DAN and the DMN is an index of efficient cognitive processing [51,52]. Thus, in the context of conscious awareness, between-network anticorrelations may reflect an effective capacity to switch between an external-oriented and an internal-oriented mode of function [20]. Furthermore, in one of our previous studies, dysfunctional interactions between the DAN and DMN were also observed in patients with amnestic mild cognitive impairment and Alzheimer’s disease (AD) [53]. Considering that memory decline is core symptom of AD and episodic memory is impaired during midazolam administration, we speculated that midazolam administration could simulate neuropsychological changes in cognitive impairment and AD. Based on this, brain regions responsible for episodic memory and abnormal neural pathways involved in episodic memory for AD could be investigated in depth. It is worth noting that cortical GABA levels change in the human brain across the lifespan [54,55]. As a member of the class of benzodiazepines, midazolam binds to the benzodiazepine site on GABA-A receptors and enhances the effects of GABA, leading to neuronal inhibition in the central nervous system [56]. Therefore, caution needs to be taken when generalizing the current results to individuals outside the age range in this study, especially generalizing to the elderly. Based on recent evidence, GABA-A receptor availability was significantly higher in the cortex of the elderly as compared with young adults [55]. It was speculated that functional connectivity within the DAN and anticorrelation between the DAN and DMN could be decreased even more robustly in the older adults as compared with young adults during same dose of midazolam-induced light sedation.

There are two possibilities that could explain reduced between-network anticorrelations due to midazolam-induced sedation. One possible explanation is that an individual’s ability to mediate the interplay between external and internal attention is decreased by the midazolam. As mentioned above, midazolam decreased the functional connectivity among the MPFC, left IPL, and left MT, which could further disrupt the normal attentional processing of visual cues and reduce response accuracy to the external environment. The other possible explanation is that the ability to mediate the interplay between external and internal attention is preserved, but the effectiveness of mediation attempts is reduced. Given that functional connectivity within the DAN is more affected by the drug as compared with connectivity within the DMN, decreased connectivity within the DAN could thus slow down the efficiency of processing external stimuli, which would further reduce the mediation between external and internal attention. Previous studies of anesthesia in human beings and monkeys have shown that the between-network anticorrelation is affected by other hypnotic anesthetic agents, including propofol and ketamine, even at low concentrations. The anticorrelation is lost after deep sedation and is regained after recovery of consciousness [9,13,57]. Along with the decreased functional connectivity within DAN, this could be a common feature of anesthesia-induced alteration of consciousness. Additionally, the essential role of between-network anticorrelations is also well documented in cases of patients with disorders of consciousness. For example, based on one prior study, between-network anticorrelations were identified in patients who had emerged from a minimally conscious state and in healthy controls, but not in patients with disorders of consciousness, suggesting the importance of orientating between the DAN and DMN for consciousness [28]. Moreover, in normal levels of consciousness, increased metabolism was associated with increased between-network anticorrelations, which seems to be of metabolic neuronal origin [28]. Taken together, our results agree with numerous previous studies that found that the ability to switch between external and internal awareness (subserved by DAN–DMN anticorrelations) supports normal consciousness states. As a result, along with the decreased functional connectivity within DAN, DAN–DMN anticorrelation might be a reliable and sensitive marker to track alterations in consciousness.

Some limitations of this study need to be mentioned. First, the sample size in the current study was relatively small, and a larger sample may detect alterations with smaller effect sizes. A sample size of 20 subjects is generally considered acceptable in such studies for detecting large effects [58] and our sample size was in the range of most published fMRI studies investigating the effect of sedation. In addition, our results replicated the reliable findings from previous reports, and strict one-sample masks were employed to examine the effects of drug injection in an effort to reduce false positives. Therefore, we are reasonably confident about the observed significant effects. Future studies should increase the sample size to reveal drug effects that may have smaller effect sizes. Second, a multiple-seed ROI approach was used in this study to examine functional connectivity during light sedation. This might produce a bias in favor of findings from previous studies because the ROIs were defined based on the literature available. Nonetheless, accumulating evidence now supports that this method can produce an acceptable degree of reproducibility [35]. This method for each network additionally limits the risk of selection bias, as it includes well-identified hubs of each network [59]. Third, during the experiment, the sedation level of all subjects was only estimated. Detailed assessments of cognitive functions, laterality, psychiatric disorders, and sleep disturbances, which are necessary and important to evaluate the sedative effects of midazolam on the brain, were not performed. Thus, we could not rule out the potential bias derived from some of the abovementioned factors. Future studies should pay great attention to the importance of neuropsychological assessment. Fourth, due to technical limitations, the online cardiorespiratory data were not recorded and included in the data analysis. However, we regressed out BOLD signal variation that could be induced by physiological fluctuations. Given that fluctuations of respiratory and heart rate related to BOLD signal at resting-state [60,61], our results could potentially be improved by independent acquisition of physiological data instead of relying on indirect markers of physiological fluctuations derived from the BOLD data. Finally, anesthesia-induced alterations of functional connectivity are dynamic over time when consciousness is diminished [62,63]. This study only investigated the altered static functional connectivity of the DAN during midazolam-induced light sedation. Future studies including participants who have submitted to stepwise increments in anesthetic agent concentration up to loss of responsiveness are expected to provide a more comprehensive understanding of the dynamic changes in DAN-related functional connectivity patterns.

## 5. Conclusions

In conclusion, the current study provides evidence that midazolam-induced light sedation is associated with reduced connectivity within the DAN and anticorrelation between the DAN and subregions of DMN. These findings deepen our understanding of the neural mechanism of sedation, and such functional patterns might have clinical implications in other medical conditions related to consciousness.

## Data Availability

The dataset included in this analysis has been previously published by Liang et al. [26].

## Supplementary Materials

The following are available online at www.mdpi.com/article/10.3390/brainsci11081107/s1, including other results and findings that are not included in the main contents. Figure S1: Seed-based functional connectivity for pre-injection section was used to derive spatial masks for positive SMN connectivity (shown in warm yellows) and negative SMN connectivity (shown in blue), Figure S2: Spatial differences in functional connectivity associated with DAN, Figure S3: Differences in connectivity scores of the “pre_MZ-post_MZ” among the brain regions that showed significant functional connectivity changes by midazolam injection between male and female, Figure S4: Spatial differences in amplitude of the low-frequency fluctuation within the SMN by midazolam injection, Table S1: Altered functional connectivity by midazolam injection, Table S2: Altered amplitude of the low-frequency fluctuation by midazolam injection.
