# Supplementary material for: The Effect of Light Sedation with Midazolam on Functional Connectivity of the Dorsal Attention Network
Source: Brain Sci. 2021 Aug 22;11(8):1107. doi: 10.3390/brainsci11081107 (PMC8392174; doi:10.3390/brainsci11081107)
Supplement: Supplementary file 1 [file brainsci-11-01107-s001.zip › brainsci-1270225-supplementary.pdf]

Supplementary Materials: The Effect of Light Sedation with Midazolam on Functional Connectivity of the Dorsal Attention Network

Junkai Wang [1], Yachao Xu [2], Gopikrishna Deshpande [3,4,5,6,7,8,9,10], Kuncheng Li [11,12], Pei Sun [1,*] and Peipeng Liang [3,4,*]

Table S1. Altered functional connectivity by midazolam injection.

Network	Brain Regions	BA	Side	Cluster Size	T Value	Peak MNI Coordinates		
						X	Y	Z
**SMN**								
	PoCG/PreCG	1/3	L	41	3.73	−40	−30	58
	PoCG/PreCG	1/3	R	28	3.77	32	−36	62
**DAN**								
	IPL	40	L	23	3.68	−44	−48	52
	MT	37	L	53	4.04	−54	−62	−18
**DMN**								
	mPFC	10	L	21	3.61	−2	60	16
	SFG	9	R	43	4.34	26	46	36
	STG	21	R	33	4.08	52	−28	−6

Abbreviations: R = Right, L = left. MNI: Montreal Neurological Institute; BA: Brodmann areas; SMN, sensorimotor network; DAN, dorsal attention network; DMN, default mode network; PoCG, postcentral gyrus; PreCG, precentral gyrus; IPL, inferior parietal lobule; MT, middle temporal area; mPFC, medial prefrontal cortex; STG, superior temporal gyrus; SFG, superior frontal gyrus.

Table S2. Altered amplitude of the low-frequency fluctuation by midazolam injection.

Network	Brain Regions	BA	Side	Cluster Size	T Value	Peak MNI Coordinates		
						X	Y	Z
**SMN**								
	PoCG/PreCG	1/3	L	90	5.12	−39	−15	45
	PoCG/PreCG	1/3	R	228	5.84	15	−33	69
	SMA	6	R	80	5.54	3	−21	63

Abbreviations: R = Right, L = left. MNI: Montreal Neurological Institute; BA: Brodmann areas; SMN, sensorimotor network; PoCG, postcentral gyrus; PreCG, precentral gyrus; SMA, supplementary motor area.

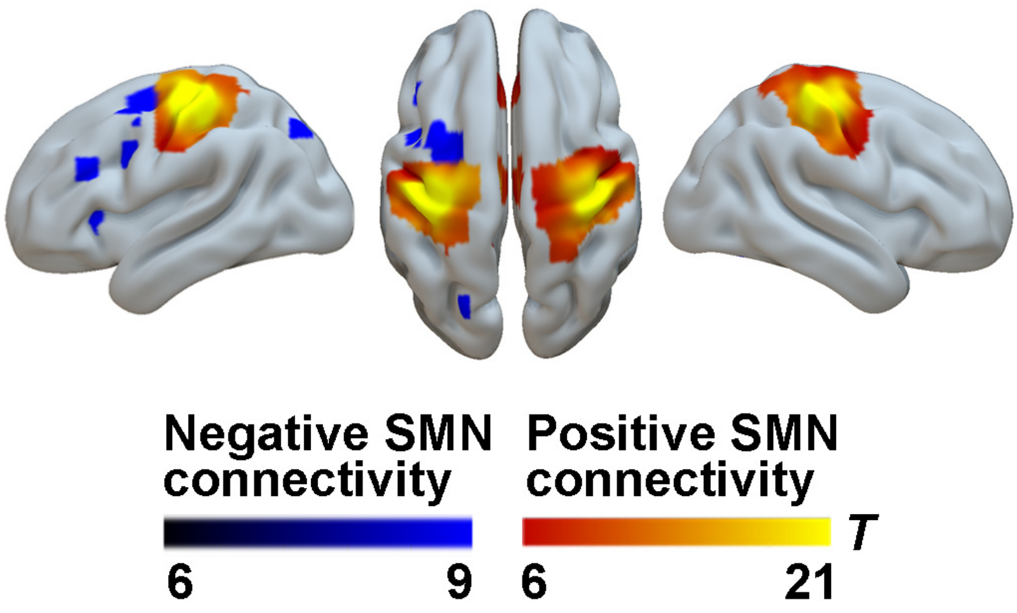

Figure S1. Seed-based functional connectivity for pre-injection section was used to derive spatial masks for positive SMN connectivity (shown in warm yellows) and negative SMN connectivity (shown in blue).

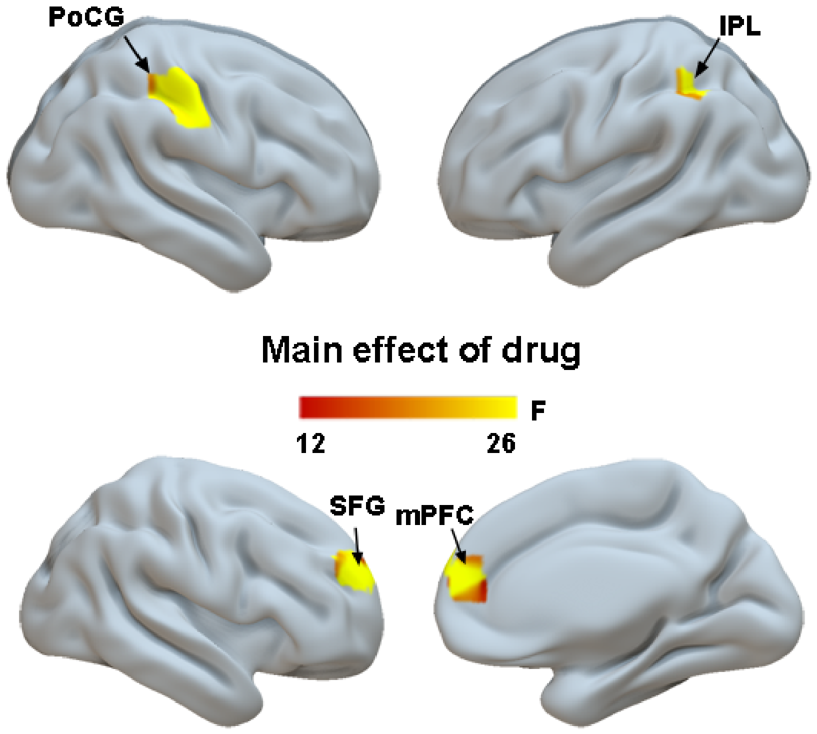

Figure S2. Spatial differences in functional connectivity associated with DAN. Two-way repeated-measures ANOVA revealed that significant main effect of drug for the DAN connectivity was identified.

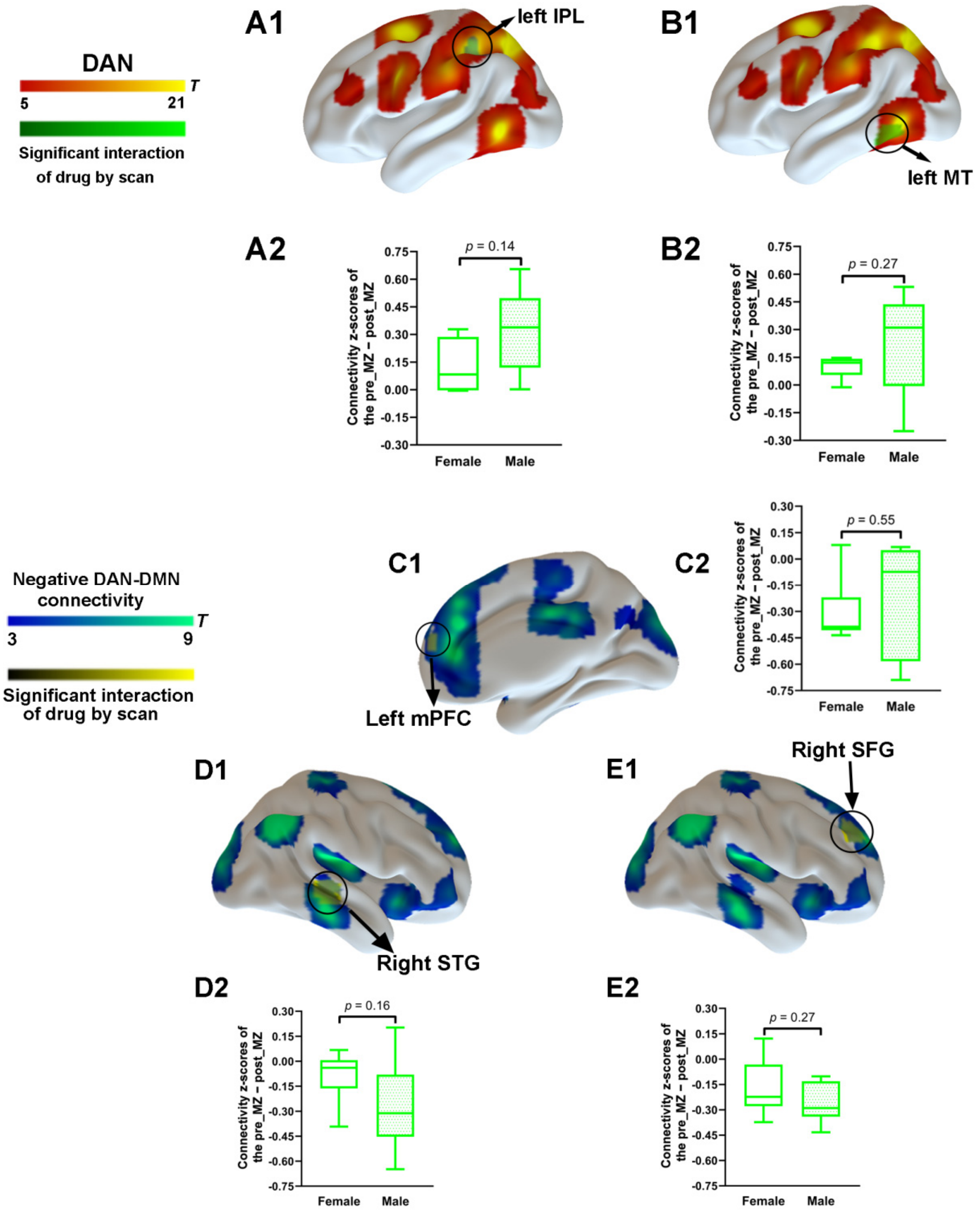

Figure S3. Differences in connectivity scores of the "pre_MZ − post_MZ" among the brain regions which showed significant functional connectivity changes by midazolam injection between male and female. Connectivity scores of the "pre_MZ − post_MZ" within the left IPL (A) and left MT (B) of the DAN that showed no significant difference between male and female. Connectivity scores of the "pre_MZ − post_MZ" between the left mPFC (C), right STG (D), right SFG (E) and the DAN that showed no significant difference between male and female. Boxplot whiskers indicate min and max. Box is defined by 25th percentile, median and 75th percentile. Abbreviations: IPL, inferior parietal lobule; MT, middle temporal area; mPFC, medial prefrontal cortex; STG, superior temporal gyrus; SFG, superior frontal gyrus; DAN, dorsal attention network; MZ, midazolam; SA, saline.

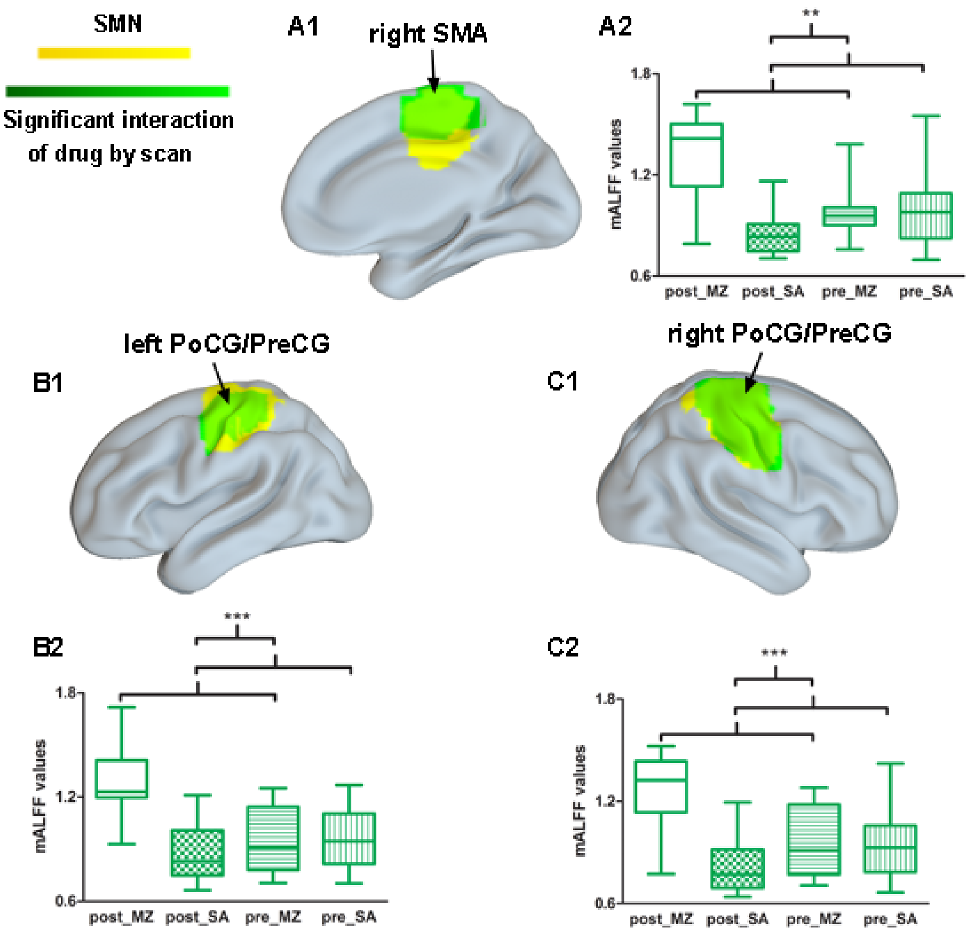

Figure S4. Spatial differences in amplitude of the low-frequency fluctuation within the SMN by midazolam injection. Two-way repeated-measures ANOVA revealed significantly increased mALFF (in green) by midazolam injection in the right SMA (A1), left postcentral gyrus and precentral gyrus (B1), and right postcentral gyrus and precentral gyrus (C1). Significantly results were overlaid on the spatial maps (shown in yellow) of the SMN (cluster-level FWE correction). (A2) Average mALFF values of right SMA that showed significant interaction between drug and injection among subjects under each condition. (A2) Average mALFF values of left postcentral gyrus and precentral gyrus that showed significant interaction between drug and injection among subjects under each condition. (B2) Average mALFF values of right postcentral gyrus and precentral gyrus that showed significant interaction between drug and injection among subjects under each condition. Boxplot whiskers indicate min and max. Box is defined by 25th percentile, median and 75th percentile. Bars represent average mALFF values and error bars indicate standard error of the means (SEM) derived from the ANOVA test on the cluster that showed significant interaction between drug and injection. ***$P < 0.001$; **$P < 0.01$. Abbreviations: SMA, supplementary motor area; PoCG, postcentral gyrus; PreCG, precentral gyrus; SMN, sensorimotor network; MZ, midazolam; SA, saline.

Supplemental Methods

MRI data acquisition

Imaging data were acquired using a Siemens Trio 3-Tesla scanner (Siemens, Erlangen, Germany). Participants were instructed to keep their head still and their eyes closed and to refrain from thinking about anything in particular. Foam padding and headphones were used to limit head movement and minimize scanner noise. The high-resolution T1-weighted anatomical images were acquired using a multi-echo magnetization prepared rapid gradient echo (MPRAGE) sequence. The acquisition parameters were as follows: repetition time = 1600 ms, echo time = 2.25 ms, inversion time = 800 ms, flip angle = 9°, acquisition matrix = 256 × 256 × 192, voxel size = 1 × 1 × 1 mm^3. BOLD functional imaging data was obtained in an axial orientation using a T2* weighted gradient-echo echo-planar imaging (EPI) sequence with the following parameters: repetition time = 2000 ms, echo time = 31 ms, flip angle = 90°, acquisition matrix = 64 × 64, field of

view = 240 mm × 240 mm, slice thickness = 4 mm, gap = 0.8 mm, 30 slices, voxel size = 3.75 × 3.75 × 4 mm^3.

Amplitude of the low-frequency fluctuation (ALFF) analysis

To better explain the changes in the SMN and DAN connectivity patterns during midazolam-induced light sedation, we also explored the ALFF changes in the SMN and DAN. For ALFF analyses, smoothed image data was performed by using Data Processing Assistant for Resting-State fMRI (DPABI, 4.3, Advanced edition (1)). Time series linear drift was removed and several covariates were regress out including the WM signal, cerebrospinal fluid signal and Friston-24 parameters. Temporal band-pass filtering (0.01–0.1 Hz) was conducted. The filtered time series of each voxel was transformed into the frequency domain and the power spectrum was obtained. For standardization, the ALFF of each voxel was further divided by the average ALFF of all voxels in the whole brain to obtain the mALFF for each voxel (2).

statistical analysis was conducted for group differences using 2×2 two-way repeated measure analysis of variance (ANOVA) on all subjects' individual mALFF maps for pre_MZ, pre_SA, post_MZ (post midazolam administration), and post_SA (post saline administration). Two main effects, drug and injection, as well as an interaction (drug-by-injection) effect were obtained by using clusters with a minimum size of 20 voxels and an individual voxel height threshold of $P < .001$. We were only interested in the interaction effect of drug by injection which represents the true drug effect.

Supplemental References

1. Yan, C.G., Wang, X.D., Zuo, X.N., Zang, Y.F. DPABI: Data Processing & Analysis for (Resting-State) Brain Imaging. *Neuroinformatics* **2016**, 14, 339–351.
2. Zang, Y.F.; He, Y.; Zhu, C.Z.; Cao, Q.J.; Sui, M.Q.; Liang, M.; Tian, L.X.; Jiang, T.Z.; Wang, Y.F. Altered baseline brain activity in children with ADHD revealed by resting-state functional MRI. *Brain Dev* **2007**, 29, 83–91.

Supplemental Results

ALFF results

In the ALFF analysis, significant interaction effect between drug and injection were observed primarily in the right supplementary motor area and the bilateral postcentral gyrus and precentral gyrus within the SMN (Supplemental Fig. S3 and Table S2). Specifically, during midazolam injection relative to the saline treatment baseline, mALFF in aforementioned regions was significantly increased. However, for mALFF within the DAN, there is no significant interaction effect between drug and injection.
